# The risk of climate ruin

**DOI:** 10.1007/s10584-016-1846-3

**Published:** 2016-11-19

**Authors:** Oliver D. Bettis, Simon Dietz, Nick G. Silver

**Affiliations:** 1 Resource and Environment Board, Institute and Faculty of Actuaries, London, UK; 2 ESRC Centre for Climate Change Economics and Policy, Grantham Research Institute on Climate Change and the Environment, and Department of Geography and Environment, London School of Economics and Political Science, London, UK

**Keywords:** Climate System, Capital Requirement, Climate Risk, Solar Radiation Management, Cumulative Emission

## Abstract

How large a risk is society prepared to run with the climate system? This is a question of the utmost difficulty and it admits a variety of perspectives. In this paper we draw an analogy with the management and regulation of insurance companies, which are required to hold capital against the risk of their own financial ruin. Accordingly, we suggest that discussions about how much to reduce global emissions of greenhouse gases could be framed in terms of managing the risk of ‘climate ruin’. This shifts the focus towards deciding upon an acceptable risk of the very worst-case scenario, and away from how “avoiding dangerous anthropogenic interference with the climate system” has come to be framed politically. Moreover it leads to the conclusion that, in terms of greenhouse gas emissions today and in the future, the world is running a higher risk with the climate system than insurance companies run with their own solvency.

## Introduction

Risk and uncertainty are central to assessing the consequences of climate change and formulating response strategies (e.g. Kunreuther et al. 2013; IPCC 2014). One key question is: how large a risk (risk in the broad sense) is society prepared to run with the climate system? This question is at the heart of enduring debates about the appropriate level of ambition, globally, in reducing greenhouse gas emissions. It is a question of the utmost difficulty, however. For one thing, as Jones et al. (2014) concisely put it: “No universal criterion exists for a good decision, including a good climate-related decision”. Thus a wide variety of legitimate perspectives exists. For another, even if we could settle on a single perspective, a question such as this remains difficult to answer, because of the timescales, uncertainties and magnitudes of change that must be contemplated.

In this paper we seek to add a new perspective to the debate, which is to compare the risk that the world is running with the climate system, defined in terms of the risk of ‘climate ruin’, with the comparable risk that insurance companies are prepared or allowed to run with their own financial ruin. This is hence an example of an actuarial perspective on climate change. In doing so, we follow a tradition of attempting to reason about our tolerance of climate risk by examining how other risks are managed in society (e.g. Posner 2004).

In the next section we summarise the system of company management and public regulation that governs insurance companies’ risk of ruin in many countries. In Section 3 we draw the analogy with the management of climate risk, by discussing what ruin would mean in terms of climate impacts. Admittedly the analogy is far from straightforward, but we can make progress by drawing upon analyses of ruin, catastrophe and collapse in related fields of intellectual inquiry, and we set out what makes climate ruin a distinctive perspective, compared with the now dominant focus on avoiding “dangerous anthropogenic interference with the climate system” (United Nations 1992, p9). In this section, we also set a threshold for climate ruin in terms of the increase in global mean temperature above the pre-industrial level. We argue for a 4 ^∘^C threshold, based on current evidence. Section 4 shows how physical modelling can be used to estimate the probability of climate ruin as a function of cumulative greenhouse gas emissions. This forms the basis of our comparison of the risk that the world is running with the climate system and the risk that insurance companies are prepared or allowed to run with their own financial ruin. The former appears to be larger than the latter. Section 5 offers some conclusions.

## The risk of ruin for insurance companies

Insurance companies are required to hold capital against the risk of failing to meet their liabilities, in particular of failing to pay claims to their policyholders in an unusually bad year, in which there are too many claims. Bankruptcy can follow. This is known in the industry as the ‘risk of ruin’. An insurer has to calculate how much capital it needs to hold in order to reduce the probability of ruin below an acceptable level. This threshold is either set by the regulator, or at a level that assures policyholders and investors the insurance company is safe.

For example, the UK’s Financial Conduct Authority (FCA) sets the capital requirement such that the risk of ruin is no more than 1 in 200 (i.e. 0.5 %) over a one-year time horizon (FSA 2008). This is the same probability that sets the capital requirement in the EU’s new Solvency II Directive (Swain and Swallow 2015). In practice, insurance companies normally hold sufficient capital such that the risk of ruin is far lower than this level. Large reinsurance companies such as Munich Re and Swiss Re typically aim for a credit rating in the region of AA. An estimate of the average default probability for corporations rated AA over a one-year horizon is currently 0.02 % or 1 in 5000 (RatingsDirect 2015).

There is a key difference between assessing capital requirements and setting premium rates for insurance policies. Setting premium rates requires estimating the mean of future claims payments (i.e. losses) arising from insurance policies. It is difficult to know what the mean loss is for any particular insurance policy, but it is not usually necessary to include a margin for prudence within the estimate, because the insurance company would normally expect to overestimate the expected loss in some cases and underestimate it in others. In contrast, the capital requirement is estimated once (usually annually) for the entire insurance company, so errors cannot be averaged out. In setting capital requirements, the focus is on the extreme right tail of the probability distribution of loss for the entire company.

The system therefore prioritises resilience to shocks, and the strategy is consistent with the pursuit of robustness and safety margins that can be found in many other areas of decision-making under uncertainty (Kunreuther et al. 2013). There is even some evidence to suggest that it has made the insurance industry more resilient to natural catastrophes such as earthquakes and hurricanes (Best 2014; Massey et al. 2003), although a convincing demonstration of cause and effect has yet to be made.

## Climate ruin

Whereas ruin of an insurance company is relatively clear-cut – the company becomes insolvent – what might ruin mean in the context of climate change? Climate ruin could mean different things at different spatial scales, but in this paper we focus on global reductions in greenhouse gas emissions. Our aim is to show how the framework can contribute to debates about global emissions targets, including attempts to evaluate whether the sum of existing efforts by countries to cut emissions is sufficient (e.g. UNEP 2015). In doing so, we adopt a perspective akin to the role of a global social planner – sometimes described as a ‘benevolent dictator’ – in economic evaluation of climate policies. That means the phenomenon of ruin that interests us occurs on a global scale, although it does not imply climate impacts fall evenly across the world. Indeed there is no reason to expect them to do so (IPCC 2014). It also means that our ultimate aim is normative in character. Investigating the risk of climate ruin that society is currently running is a means to understanding whether that risk *ought* to be reduced through further mitigation, or other strategies. For the most part we do not directly consider adaptation as a response strategy, but we must make implicit assumptions about it in order to judge how damaging emissions will be.

A representative dictionary definition of ruin is “The state or condition of a ... society which has suffered decay or downfall” (Oxford English Dictionary 2014). This implies attention should focus on the magnitude of climate change that triggers severe negative impacts, and that those impacts must affect the economic and social domains, but it still leaves much to be clarified. As a means of sharpening our understanding of what constitutes climate ruin, it is worth reviewing comparable notions of ruin, catastrophe and collapse in different fields of research.

### Catastrophes and disasters in economics

There has periodically been strong interest in economics in rare disasters and catastrophes, and their implications. To take a prominent recent example, Barro (2006) has argued that acknowledging the existence of rare economic disasters can reconcile the predictions of standard theory about asset prices with observations. Barro’s definition of a rare economic disaster, which is what is of interest here, is quite natural for an economist: a sharp contraction in income/output per capita. In particular, he looks at instances during the 20th century when a country’s real GDP per capita fell by 15 per cent or more over a period of three to eight years. The main causes of these contractions were World Wars I and II, and the Great Depression.

An exceptionally large contraction in income per capita is the natural definition of ruin in economics, because income per capita is the benchmark measure of living standards, individual well-being and social welfare. Accordingly, this is the principal way in which the spectre of catastrophe has been considered in the economics of climate change (Martin and Pindyck 2015; Weitzman 2009, 2012), but with three important differences. First, the spatial scale of analysis shifts from the country to the globe. Second, economic models of climate change use a broader definition of income per capita, where it serves as an equivalent measure of individual well-being, something that is not directly observed. This means that mortality, among other things, can be included, so ruin can mean loss of life, as well as loss of income. Third, the timescale over which income contracts becomes less clear, but in general the analysis extends over at least a century and sees a climate catastrophe as unfolding over decades, not just years. On the other hand, as Posner (2004) points out, the rate of physical change cannot be so slow that adaptation eliminates the risk of social and economic ruin. In quantifying economic catastrophe in terms of income per capita and mortality, and looking into the long-run future, these recent analyses are close in spirit, if certainly not in methodology, to the *Limits to Growth* series that began with Meadows et al. (1972).

### Collapse of historical civilisations

An extensive body of research has studied instances of the collapse of historical civilisations, and the reasons for them. Examples, of which there are many (Motesharrei et al. 2014), include the Roman Empire in Europe, Maya civilisation in Central America, and Khmer Empire in Southeast Asia. While there is naturally a strong overlap with economists’ measures of collapse, in that collapsing civilisations usually experience large declines in economic activity and increases in mortality, the focus of this work is nonetheless distinctive. For our purposes, it adds political and social dimensions. In addition, since historical civilisations tended to collapse over decades, not years, it extends our time horizon in line with the notion of a climate catastrophe in economics, and away from transitory economic recessions in the 20th century.

According to Tainter, a “society has collapsed when it displays a rapid, significant loss of an established level of sociopolitical complexity” (Tainter 1988, p4). Diamond (2005) adopts a similar definition of “a drastic decrease in human population size and/or political/economic/social complexity” (p3). Therefore collapse is, as Tainter puts it, “fundamentally a matter of the sociopolitical sphere” (p4). Measures of a collapse of sociopolitical complexity include: reduced social stratification and differentiation; reduced economic specialisation; a breakdown in centralised control and the rule of law; reduced flows of goods, services and information; reduced investment in monumental architecture, and so on. Some of these phenomena were certainly experienced during Barro’s (2006) rare economic disasters of the 20th century, in particular in European countries during and immediately after the two World Wars. In other countries in his data set, however, disaster was not characterised by the sheer loss of sociopolitical complexity found in the collapsing civilisations of the pre-industrial world. Nonetheless, the modern world clearly is capable of generating collapses, such as Rwanda and Somalia (Diamond 2005).

### Climate thresholds and tipping elements

In climate research, concern about the prospect of catastrophe and collapse has been a primary motivation for analysing physical thresholds in the climate system. These are sometimes described as tipping points that correspond with ‘tipping elements’ of the system (Lenton et al. 2008), or in other words ‘large-scale singular events’ (IPCC 2014). The worry is that crossing these tipping points would bring about abrupt climatic and environmental change. Instances of abrupt climate change can be found in both the instrumental and paleoclimatic records, such as the Dust Bowl drought and the Younger Dryas cold event respectively (see National Research Council Committee on Abrupt Climate Change 2002; Alley et al. 2003). Not all tipping points/elements are likely to be associated with abrupt climate change, but some are, in that they have a transition timescale of years or decades (Lenton et al. 2008).

A feature of this work is that it has remained largely focused on the physical phenomena in question, with some work on related abrupt change in ecosystems, rather than the social and economic consequences of crossing tipping points (Keller et al. 2008; Lenton 2011). There is very little work that does the latter, especially in a comprehensive manner, simply due to the difficulties involved in modelling the consequences of abrupt change formally. What does exist appears to be within the realm of Integrated Assessment Modelling, which is a relatively flexible medium. In IAMs, the characteristic approach to incorporating tipping points is via a reduced form. For example, Keller et al. (2004) represent shutdown of the Atlantic Thermohaline Circulation in the DICE model as a step increase in global GDP losses as a function of crossing a threshold in the atmospheric concentration of greenhouse gases. Similarly, Lemoine and Traeger (2014) introduce tipping points to the equilibrium climate sensitivity parameter and the removal of atmospheric CO _2_ in DICE, while Whiteman et al. (2013) add a large pulse of methane, released from melting permafrost, to the standard PAGE model. Climate ruin in these studies is hence an economic phenomenon, modelled in the abstract.

### Dangerous climate change

The process of giving meaning to “dangerous anthropogenic interference with the climate system”, introduced by Article II of the United Nations Framework Convention on Climate Change (UNFCCC; United Nations, 1992), is also clearly of relevance, given the common-sense similarities between the notions of dangerous climate change (Dessai et al. 2004) and climate ruin. The 2009 Copenhagen Accord recognised an existing line of thought, which can be traced back at least as far as a European Union decision in 1996 (Council of the European Union 1996), that 2 ^∘^C marks the threshold for dangerous anthropogenic interference (Randalls 2010). The Paris Agreement goes beyond this by including the stated aim of “holding the increase in the global average temperature to well below 2 ^∘^C ... and pursuing efforts to limit the temperature increase to 1.5 ^∘^C above pre-industrial levels” (United Nations 2015).

The difficulty is that it has always been unclear what risk is being tolerated of missing the 2 ^∘^C (or 1.5 ^∘^C) threshold for dangerous anthropogenic interference. The correspondence between a given emissions path and warming is uncertain. Moreover, the political process has been unable to give clarity on what would constitute an acceptable probability of missing the target, a lack of clarity that is further diminished by the disconnect between the stated aim to hold temperatures down and pledged emissions reductions at the Copenhagen and Paris Conferences of the Parties to the UNFCCC (den Elzen et al. 2011; UNEP 2015). Critics have accused the 2 ^∘^C target of being an exercise in political obfuscation (Victor and Kennel 2014). At the very least it seems clear that the UN temperature targets have not closed the debate about how much to reduce emissions.

### Summary: what is climate ruin and when might it be triggered?

We might simply treat climate ruin and dangerous climate change as being interchangeable concepts, and therefore adopt the 2 ^∘^C or even 1.5 ^∘^C targets as a threshold for climate ruin. This approach cannot be dismissed out of hand, yet it is doubtful that the evidence supports it, because, unlike the meaning of dangerous climate change in politics, our definition of climate ruin is a worst-case scenario at the global level. This is not only the strongest analogy with ruin of insurance companies, where it is an existential risk to the company, it is also consistent with how ruin is conceived in the literatures surveyed above. These literatures depict rapid – but still multi-decadal – breakdown of economic activity, human health and political/social order and complexity.

We can evaluate the recent contribution of Working Group II to the IPCC’s *Fifth Assessment Report* in this light. It revives the Panel’s tradition of summarising the impacts of rising temperatures with five ‘reasons for concern’ (IPCC 2014). At 2 ^∘^C above the pre-industrial level, IPCC classifies the level of three of the five key risks (i.e. reasons for concern) as high: the risks to unique and threatened systems, the risks of extreme weather events, and the risks for disproportionately affected people and communities. On the other hand, the risks of global aggregate impacts and the risks of large-scale singular events are moderate. At 4 ^∘^C above the pre-industrial level, all five key risks are high and in the case of unique and threatened systems they are very high. If we think of what environmental, economic and social impacts are consistent with a worst-case scenario at the global level, then it can be argued that the risks of global aggregate impacts and of large-scale singular events are key. On the basis of the IPCC’s reasons for concern then, we suggest linking climate ruin with no fewer than 4 ^∘^C of warming (also see New et al. 2011; Schellnhuber et al. 2012).

Before moving on, it is important to point out that doing so appears to be inconsistent with the evidence presented in the majority of economic IAMs. Most of these models do not forecast large impacts of climate change until the global mean temperature reaches an exceedingly high level. At 4 ^∘^C above pre-industrial, standard versions of the three leading IAMs estimate impacts equivalent to a loss of global GDP of about 1–5 % (Interagency Working Group on Social Cost of Carbon 2010). If the global economy grows as it tends to at several per cent *per year*, this clearly constitutes modest damages. However, the damage forecasts of IAMs, in particular at high temperatures, have been criticised for being entirely driven by assumptions that cannot currently be constrained by data (Pindyck 2013; Revesz et al. 2014). Some have further argued that these assumptions are inconsistent with other impacts research and are thus implausible (Stern 2013; Weitzman 2012). Consequently alternative assumptions have been proposed, which generate much larger damages at 4 ^∘^C above pre-industrial (Dietz and Stern 2015; Weitzman 2012). Ultimately the damage functions in IAMs can be made to represent any assumptions and we therefore doubt whether the evidence from these models is sufficiently strong at this time to justify an alternative threshold for climate ruin.

## Emissions limits to avoid climate ruin

The risk of ruin in the insurance industry applies year to year, because companies can adjust premia and vary capital holdings on this timescale, i.e. it is assumed that they are not locked into positions requiring resilience to be evaluated over a longer period. By contrast, the global mean temperature depends on the atmospheric concentration of greenhouse gases and therefore cumulative carbon emissions over centuries, i.e. our position is significantly locked in. This makes the choice of time horizon in analyses of the impacts of climate change a thorny, if often neglected, issue. Many assessments are truncated at the end of the 21st century, but the atmospheric residence time of CO _2_ justifies a much longer-term view. We take our objective to be to control emissions so as *never* to exceed the given probability of climate ruin (i.e. we focus on peak warming, rather than transient or equilibrium warming *per se*).

Table 1 reports estimates of the probability of exceeding 4 ^∘^C warming above pre-industrial as a function of cumulative carbon emissions since pre-industrial from the one major study to so far report these explicitly (Zickfeld et al. 2009). These estimates are generated from an ensemble of simulations of an Earth System Climate Model. The min-max range is generated from a range of probability density functions of the climate sensitivity, coupled with two deterministic assumptions about the strength of the climate-carbon cycle feedback. Since linking climate ruin with 4 ^∘^C warming is not beyond dispute, we also report estimated probabilities of exceeding 2–3 ^∘^C warming. We cannot report probabilities of exceeding 5 ^∘^C warming or more, because the underlying study does not report them either.

**Table 1:** Estimates from Zickfeld et al. (2009) of the probability of exceeding 2–4 ^∘^C warming above pre-industrial as a function of cumulative carbon emissions since pre-industrial (gigatonnes of carbon)

Cumulative carbon	2 ^∘^C		3 ^∘^C		4 ^∘^C	
emissions (GtC)	min.	max.	min.	max.	min.	max.
500	0.03	0.5	0	0.34	0	0.27
1000	0.34	0.8	0.03	0.51	0	0.4
1500	0.63	0.98	0.23	0.73	0.05	0.53
2000	0.81	1	0.48	0.89	0.18	0.69
3000	0.92	1	0.75	1	0.53	0.91
4000	0.95	1	0.87	1	0.71	0.99

Before drawing conclusions from Table 1, it is important to highlight the limitations of the notion of probability in this setting, where the degree of correspondence between the climate model on which the analysis is based and the real climate system is unknown (Stainforth et al. 2007). There is in other words no guarantee these model probabilities correspond with the real probability of the climate system warming 2–4 ^∘^C in response to a given pulse of cumulative carbon emissions. At the same time, the degree of bias is essentially unknowable. The fact that Zickfeld et al. (2009) report a range of probabilities illustrates the probabilities themselves are uncertain.

With this caveat in mind, let us compare the probability of climate ruin in Table 1 with the probability that insurance companies are prepared or allowed to run with their own solvency. Recall from Section 2 that insurers’ risk of ruin has been capped by industry regulators in many countries at 0.5 % over one year, which amounts to 40 % over 100 years, a more reasonable timescale for comparison with the probability of climate ruin. But the actual risk appetite of insurers is usually lower. Companies seeking an AA rating will face a risk of ruin of approximately 0.02 % over one year, which is 2 % over 100 years. By comparison, Table 1 shows that the probability of peak warming of 4 ^∘^C may be as high as 27 % even for historical cumulative emissions, which are of the order of 500GtC. The probability increases significantly as cumulative emissions rise beyond 500GtC. IPCC suggests that, along a baseline emissions scenario, 1000GtC will have been emitted cumulatively before 2060 with certainty (Clarke et al. 2014). The same analysis shows that there is about a 50 % chance of cumulative emissions reaching 2000GtC by the end of the century, which as Table 1 shows is associated with a probability of 4 ^∘^C warming of 18–69 %. According to Rogelj et al. (2016), implementation of the pledges made at COP21 in Paris (and continuing at a similar level of ambition after 2030) will most likely result in cumulative emissions in the region of 1200GtC by 2100, with the lowest estimate being around 900GtC.

Although they do not report explicit estimates of the probability of 4 ^∘^C warming, data reported in Allen et al. (2009) can be used as a basis for producing such estimates and thus provide a point of partial comparison. These estimates are generated from an ensemble of a simple coupled climate-carbon cycle model, with uncertainty about five physical parameters. According to our own fit of Allen et al. (2009, Fig. 3) cumulative carbon emissions of 1000 GtC since pre-industrial will lead to 4 ^∘^C warming with a probability of 16 %. This is roughly in the middle of the range reported by Zickfeld et al. (2009).

## Conclusions

The aim of this paper has been to augment our understanding of the level of risk (risk in the broad sense) that society is running with the climate system, based on historical and likely future emissions of greenhouse gases. The novelty has been in reasoning about this by analogy with the insurance industry, which holds capital against the risk of ruin, a strategy to ensure resilience against shocks. Contingent on setting the threshold for climate ruin at 4 ^∘^C warming above the pre-industrial level, it is clear that society is currently running a larger risk with the climate system than insurance companies are prepared or allowed to run with their own solvency. This is even clearer, if climate ruin is expected to be triggered by less than 4 ^∘^C warming.

Attention naturally turns to what one should conclude from the comparison. On the one hand, it might be argued that there are legitimate reasons why society tolerates a greater amount of climate risk. One such reason might be that climate risk is costlier to reduce than the risk of ruin for insurance companies, which is essentially their cost of solvency capital. Another, related reason is that global catastrophe risks like climate ruin might not be governable in the same way as an insurance company can relatively easily manage its risk of ruin. From the point of view of a global social planner, the risk of nuclear war that was run during the Cuban Missile Crisis, for instance, may well have been unacceptable, but the key protagonists were not acting like a global social planner, which is of course a fictitious concept.

On the other hand, it might be argued that the comparison reinforces the case for greater ambition in reducing greenhouse gas emissions globally. Indeed, a strict interpretation of the modelling data in Table 1, reinforced by Allen et al. (2009), would be that (net) emissions reductions need to be exceptionally deep, in order to bring the risk of climate ruin down to a level comparable with the risk of ruin for insurance companies. Geoengineering technologies that remove carbon dioxide from the atmosphere may be required. Indeed, if climate ruin does occur at 4 ^∘^C above pre-industrial, and efforts to reduce net emissions prove unsuccessful, a case might also be made to pursue solar radiation management (Keith 2013), although it has been questioned whether geoengineering technologies as a whole are politically feasible, effective in regulating climate, or safe (Vaughan and Lenton 2011; Barrett et al. 2014). The other response strategy in the face of climate ruin is of course adaptation. The idea of climate ruin implies adaptation would need to be ‘transformational’, defined as adaptations “that are adopted at a much larger scale or intensity, those that are truly new to a particular region or resource system, and those that transform places and shift locations” (Kates et al. 2012). As IPCC makes clear, adaptation and mitigation are not wholly substitutable, rather limits to adaptation mean that the two are partly complementary strategies (IPCC 2014). Therefore it would seem the most that transformational adaptation could achieve in this setting is a partial reduction in necessary reductions in net carbon emissions.
